# COVID-19 vaccination willingness among people living with HIV in Shijiazhuang, China: a cross-sectional survey

**DOI:** 10.3389/fmed.2024.1322440

**Published:** 2024-01-17

**Authors:** Xihong Zhang, Haoting Zhan, Lijing Wang, Yongmei Liu, Xinru Guo, Chen Li, Xiaomeng Li, Beilei Li, Haolong Li, Yingxia Li, Qian Chen, Huixia Gao, Fumin Feng, Yongzhe Li, Erhei Dai

**Affiliations:** ^1^Center for Disease Control and Prevention of Yunyan District in Guiyang, Guiyang, China; ^2^Department of Clinical Laboratory, State Key Laboratory of Complex, Severe and Rare Diseases, Peking Union Medical College Hospital, Chinese Academy of Medical Science and Peking Union Medical College, Beijing, China; ^3^Department of AIDS, The Fifth Hospital of Shijiazhuang, North China University of Science and Technology, Shijiazhuang, China; ^4^School of Public Health, North China University of Science and Technology, Tangshan, China; ^5^Department of Medical Research Center, Peking Union Medical College Hospital, Chinese Academy of Medical Science and Peking Union Medical College, Beijing, China; ^6^Hebei Key Laboratory of Immune Mechanism of Major Infectious Diseases and New Technology of Diagnosis and Treatment, The Fifth Hospital of Shijiazhuang, North China University of Science and Technology, Shijiazhuang, China; ^7^Department of Clinical Laboratory, Peking Union Medical College Hospital, Beijing, China

**Keywords:** people living with HIV, COVID-19, vaccine, willingness, influencing factors

## Abstract

**Objectives:**

The COVID-19 pandemic imposed an enormous disease and economic burden worldwide. SARS-CoV-2 vaccination is essential to containing the pandemic. People living with HIV (PLWH) may be more vulnerable to severe COVID-19 outcomes; thus, understanding their vaccination willingness and influencing factors is helpful in developing targeted vaccination strategies.

**Methods:**

A cross-sectional study was conducted between 15 June and 30 August 2022 in Shijiazhuang, China. Variables included socio-demographic characteristics, health status characteristics, HIV-related characteristics, knowledge, and attitudes toward COVID-19 vaccination and COVID-19 vaccination status. Multivariable logistic regression was used to confirm factors associated with COVID-19 vaccination willingness among PLWH.

**Results:**

A total of 1,428 PLWH were included, with a 90.48% willingness to receive the COVID-19 vaccination. PLWH were more unwilling to receive COVID-19 vaccination for those who were female or had a fair/poor health status, had an allergic history and comorbidities, were unconvinced and unsure about the effectiveness of vaccines, were unconvinced and unsure about the safety of vaccines, were convinced and unsure about whether COVID-19 vaccination would affect ART efficacy, or did not know at least a type of domestic COVID-19 vaccine. Approximately 93.00% of PLWH have received at least one dose of the COVID-19 vaccine among PLWH, and 213 PLWH (14.92%) reported at least one adverse reaction within 7 days.

**Conclusion:**

In conclusion, our study reported a relatively high willingness to receive the COVID-19 vaccination among PLWH in Shijiazhuang. However, a small number of PLWH still held hesitancy; thus, more tailored policies or guidelines from the government should be performed to enhance the COVID-19 vaccination rate among PLWH.

## 1 Introduction

It has been approximately 3 years since the coronavirus disease 2019 (COVID-19) pandemic, caused by severe acute respiratory syndrome coronavirus 2 (SARS-CoV-2), which has imposed an enormous disease and economic burden worldwide. Although herd immunity could also be established through natural SARS-CoV-2 infection, it may have devastating consequences for humans (1). A mass COVID-19 vaccination is conducive to induced herd immunity, decreasing morbidity and mortality (2) and is also one of the most cost-effective health interventions to control the COVID-19 pandemic (3, 4). Many clinical trials and real-world studies have shown that COVID-19 vaccines have favorable safety and immunogenicity and are obviously effective in preventing SARS-CoV-2 infection and improving severe disease outcomes in the general population (5–9).

Immunosuppressed individuals may be at higher risk of severe COVID-19 outcomes (10). The human immunodeficiency virus (HIV) primarily attacks the CD4^+^T cells of the human immune system, resulting in weakened defenses against many infections and virus-related cancers (11). Previous studies have reported that, compared with HIV-negative individuals, people living with HIV (PLWH) have an increased risk of becoming ill with COVID-19 (12), intubation, and in-hospital death rates (13, 14), especially in those with unsuppressed HIV viral replication or lower CD4^+^T cell counts (15, 16). Moreover, a systematic review indicated that the COVID-19 pandemic impeded access to follow-up, care, and treatment services for PLWH (17), which may result in a greater mental and disease burden for PLWH. Hence, the timely and effective implementation of public health measures to control COVID-19 could be of great benefit to PLWH.

While COVID-19 vaccination is crucial to prevent the severity and lethality of the disease caused by SARS-CoV-2 infection and control the outbreak (4, 18), achieving a high level of vaccination coverage also requires consideration of people’s willingness to be vaccinated, in addition to assessing the effectiveness and safety of the vaccine. Based on previous research, 68.4% of the global population is willing to receive the COVID-19 vaccination (19), and the acceptance of the COVID-19 vaccine varied extensively among different countries, ranging from 54.85% in Russia to 88.62% in China (20). Among PLWH, studies in China (which included eight cities but not Shijiazhuang) (21) and in the Middle East and North Africa region (22) have shown that the proportion of people willing to receive the COVID-19 vaccination was 57.2 and 64.6%, respectively. Therefore, to achieve early herd immunization, it is important to understand people’s willingness to receive the COVID-19 vaccination.

As the closest provincial capital city to the Chinese capital, Shijiazhuang has an important strategic position. There are no relevant studies to evaluate COVID-19 vaccination willingness among PLWH in Hebei Province. Based on the above considerations, this study aimed to investigate COVID-19 vaccination willingness and influencing factors among PLWH in Shijiazhuang so as to make tailored vaccination strategies for this special population.

## 2 Methods

### 2.1 Study design, setting, and participants

A cross-sectional study was conducted in the Fifth Hospital of Shijiazhuang (a tertiary referral university hospital for treating SARS-CoV-2 and HIV infections) between 15 June and 30 August 2022. All the individuals were recruited through convenience sampling. PASS 11.0 was used to calculate the minimum sample necessary, assuming a significance level of 0.05 and an allowable error of 2%, with a willingness rate (90%) of COVID-19 vaccination among PLWH (which was higher than previous studies from China (23) and the United States (24)) and a rate (20%) of invalidity considered, resulting in a minimum sample size of 1,142.

Eligible participants would be informed of the study’s purpose, risks, and benefits before completing the questionnaire, and then they would be surveyed via face-to-face interviews or an online investigation platform named Wenjuanxing (www.wjx.cn). As compensation, participants had the opportunity to get a gift worth approximately 25 RMB. The study protocol was approved by the Medical Ethics Committee of the Fifth Hospital of Shijiazhuang and Peking Union Medical College Hospital, and the need for informed consent was waived.

The eligibility criteria were as follows: (1) has a confirmed HIV infection and has been receiving the antiretroviral therapy (ART) regimen; and (2) has consented to participate in the survey. Participants would be excluded if (1) they were under 18 years old; (2) they had been infected with SARS-CoV-2; (3) they were under 180 s to complete the survey for the online version (which we judged to be the minimal reasonable time to complete the questionnaire); (4) they was more than one response per participant; and (5) they provided incorrect COVID-19 vaccination information. The official information about the COVID-19 vaccination of each patient from the CDC is double-checked with the answers from PLWH’s questionnaire. We regarded the unmatched ones as invalid questionnaires and excluded them from enrollment of this study; (6) the relevant HIV infection information of PLWH was not retrieved from the China Information System for Diseases Control and Prevention.

### 2.2 Questionnaire data sources and measurement

The questionnaire was self-designed based on previous studies (23, 25) and was endorsed by a panel of six experts with medical backgrounds. It involved five sections: socio-demographic characteristics, health status characteristics, HIV-related characteristics, knowledge, and attitudes toward COVID-19 vaccination and COVID-19 vaccination status.

Socio-demographic characteristics were collected, including age, sex, height, weight, ethnicity, marital status, education level, occupation, monthly income, and area of long-term residence. Health status characteristics included present health status, comorbidities, and previous allergic history to food/drug/vaccine/other. HIV-related characteristics were gathered from the Acquired Immune Deficiency Syndrome (AIDS) Comprehensive Prevention and Control Data Information Management System of the Chinese Center for Disease Control and Prevention (CDC), including the route of HIV transmission, last CD4^+^/CD8^+^T cells, time living with HIV, the stages of HIV infection, and the symptoms associated with HIV infection in the last 3 months. To evaluate the knowledge and attitudes toward COVID-19 vaccination, we collected beliefs about vaccine effectiveness and safety, understanding of domestic COVID-19 vaccine types, concerns about the impact of COVID-19 vaccination on ART efficacy, and the situation for proactively consulting vaccination information through medical staff. For COVID-19 vaccination status, participants were asked to answer “whether you have been vaccinated or not, the exact time and brand of each vaccine, and the local or systemic adverse reactions after vaccination within 7 days.” Finally, to assess vaccination willingness, we asked participants, “are you willing to receive COVID-19 vaccine?”

### 2.3 Bias

There was a potential bias because some of our PLWH patients had already received the COVID-19 vaccination before participating in this cross-sectional study.

### 2.4 Study size

**Figure 1:**
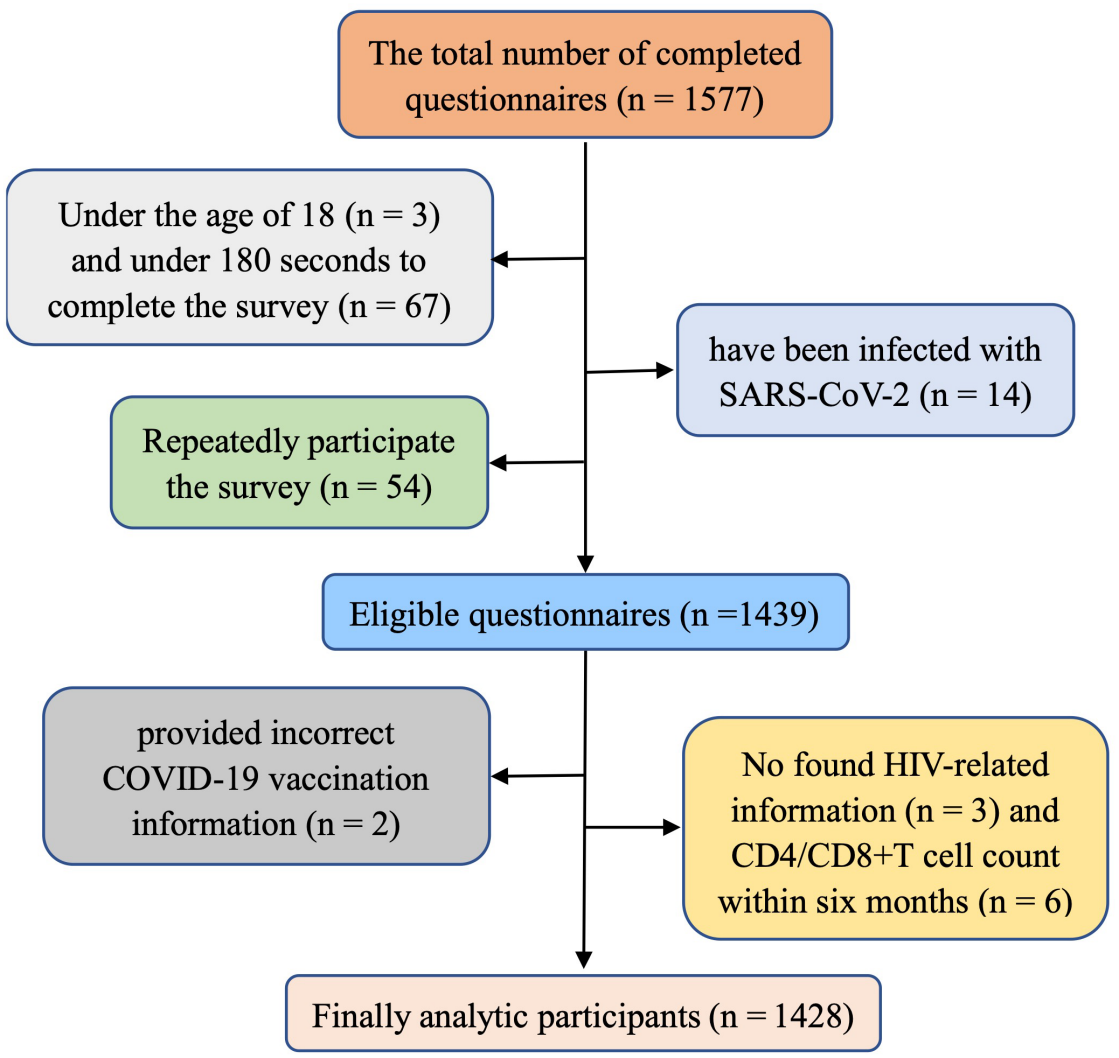
Flow chart for participants selected in the survey.

A total of 1,577 questionnaires were collected, leaving an analytic sample of 1,428 (the valid response rate was 90.55%). The selection process for valid questionnaires is shown in Figure 1.

### 2.5 Quantitative variables and statistical methods

According to COVID-19 vaccination willingness, all participants were divided into “willing” or “unwilling” to receive the COVID-19 vaccination group. The continuous variables were summarized as median and interquartile range (IQR) and tested by the Mann–Whitney U-test. The categorical variables were expressed as frequencies and percentages and compared by the chi-square test.

Subsequently, multivariable logistic regression was conducted to confirm factors associated with COVID-19 vaccination willingness among PLWH. The variables identified from the univariate analysis (*p* < 0.05) were further analyzed in the adjusted logistic regression model. The aforementioned variables associated with COVID-19 vaccination willingness were presented with crude and adjusted odds ratios (ORs) with 95% confidence intervals (CIs). A two-tailed *p*-value less than 0.05 was considered statistically significant. Statistical analyses were performed using SPSS version 25.0 (IBM, New York, United States), and the bar diagram and pie chart were visualized by GraphPad Prism 9.0 (San Diego, California, United States).

## 3 Results

### 3.1 Socio-demographic characteristics

Among 1,428 PLWH, 90.48% (1,292/1,428) were willing to receive the COVID-19 vaccination. The participants included 1,330 males (93.14%), with a median age of 40.39 years (IQR 31.78–52.18). The socio-demographic characteristics showed no statistical difference between those who are willing and unwilling to receive the COVID-19 vaccination group in the majority of variables, including age, body mass index (BMI), ethnic minorities, education level, occupation, and area of residence. However, the proportion of females, marital status (single, divorced, or widowed) and less than 5,000 RMB in monthly income was higher in the unwilling to accept the COVID-19 vaccination group (*p* < 0.05) (Table 1).

**Table 1:** Socio-demographic characteristics of all participants.

Variables	All participants (*N* = 1,428)	Willing to receive COVID-19 vaccination (*N* = 1,292)	Unwilling to receive COVID-19 vaccination (*N* = 136)	*p*-value
Age (year), median (IQR)	40.39 (31.78, 52.18)	40.50 (32.01, 52.33)	39.00 (30.73, 51.83)	0.395
<60	1,308 (91.60)	1,184 (91.64)	124 (91.18)	0.853
≥60	120 (8.40)	108 (8.36)	12 (8.82)	
**Sex, *n* (%)**				
Male	1,330 (93.14)	1,210 (93.65)	120 (88.24)	0.017
Female	98 (6.86)	82 (6.35)	16 (11.76)	
BMI (kg/m^2^), median (IQR)	22.86 (20.76, 25.15)	22.87 (20.87, 25.16)	22.04 (20.10, 24.66)	0.395
<18.5	102 (7.14)	90 (6.97)	12 (8.82)	0.105
18.5–23.9	788 (55.18)	704 (54.49)	84 (61.76)	
≥24.0	538 (37.68)	498 (38.54)	40 (29.41)	
**Ethnic minorities, *n* (%)**				
Yes	41 (2.87)	38 (2.94)	3 (2.21)	0.625
No	1,387 (97.13)	1,254 (97.06)	133 (97.79)	
**Marital status, *n* (%)**				
Married	637 (44.61)	592 (45.82)	45 (33.09)	0.004
Other (single, divorced or widowed)	791 (55.39)	700 (54.18)	91 (66.91)	
**Education level, *n* (%)**				
Primary school or below	87 (6.09)	77 (5.96)	10 (7.35)	0.823
Junior high school	393 (27.52)	356 (27.55)	37 (27.21)	
Senior high school	330 (23.11)	296 (22.91)	34 (25.00)	
College degree or above	618 (43.28)	563 (43.58)	55 (40.44)	
**Occupation, *n* (%)**				
Public official	68 (4.76)	62 (4.80)	6 (4.41)	0.346
Professional and technical personnel	139 (9.73)	120 (9.29)	19 (13.97)	
Business/service staff	126 (8.82)	115 (8.90)	11 (8.09)	
Industrial worker	159 (11.13)	144 (11.15)	15 (11.03)	
company employee	229 (16.04)	216 (16.72)	13 (9.56)	
Farmer	146 (10.22)	132 (10.22)	14 (10.29)	
Student	31 (2.17)	29 (2.24)	2 (1.47)	
Others	530 (37.11)	474 (36.69)	56 (41.18)	
**Monthly income (RMB), *n* (%)**				
<2,000	395 (27.66)	349 (27.01)	46 (33.82)	0.036
2,000–5,000	627 (43.91)	580 (44.89)	47 (34.56)	
5,001–10,000	330 (23.11)	299 (23.14)	31 (22.79)	
>10,000	76 (5.32)	64 (4.95)	12 (8.82)	
**Area of residence, *n* (%)**				
Rural	374 (26.19)	345 (26.70)	29 (21.32)	0.145
Township	133 (9.31)	115 (8.90)	18 (13.24)	
Urban	921 (64.50)	832 (64.40)	89 (65.44)	

BMI, body mass index.

### 3.2 The health status characteristics and HIV-related characteristics

The health status characteristics and HIV-related characteristics were analyzed in the PLWH who are willing and unwilling to accept the COVID-19 vaccination. Compared with the group willing to accept vaccination, the proportion of the PLWH with fair or poor health status, allergic history, and comorbidities was high in the group unwilling to accept vaccination (*p* < 0.001). The other variables did not differ between the two groups (Table 2).

**Table 2:** Health status characteristics and HIV-related characteristics of all participants.

Variables	All participants (*N* = 1,428)	Willing to receive COVID-19 vaccination (*N* = 1,292)	Unwilling to receive COVID-19 vaccination (*N* = 136)	*p*-value
**Health status, *n* (%)**				
Good	1,144 (80.11)	1,070 (82.82)	74 (54.41)	<0.001
Fair	260 (18.21)	210 (16.25)	50 (36.76)	
Poor	24 (1.68)	12 (0.93)	12 (8.82)	
**Allergic history, *n* (%)**				
Yes	231 (16.18)	189 (14.63)	42 (30.88)	<0.001
No	1,197 (83.82)	1,103 (85.37)	94 (69.12)	
**Comorbidities, *n* (%)**				
Yes	256 (17.93)	216 (16.72)	40 (29.41)	<0.001
No	1,172 (82.07)	1,076 (83.28)	96 (70.59)	
Time living with HIV (years), *n* (%)	4.33 (2.22, 6.71)	4.35 (2.30, 6.75)	4.28 (1.81, 6.21)	0.285
<1	163 (11.41)	147 (11.38)	16 (11.76)	0.701
1–5	650 (45.52)	584 (45.20)	66 (48.53)	
>5	615 (43.07)	561 (43.42)	54 (39.71)	
**Mode of HIV transmission, *n* (%)**				
Homosexual transmission	1,071 (75.00)	972 (75.23)	99 (72.79)	0.785
Heterosexual transmission	313 (21.92)	280 (21.67)	33 (24.26)	
Others/uncertain	44 (3.08)	40 (3.10)	4 (2.94)	
**The stages of HIV infection, *n* (%)**				
*I*	1,000 (70.03)	914 (70.74)	86 (63.24)	0.132
*II*	36 (2.52)	34 (2.63)	2 (1.47)	
*III*	35 (2.45)	32 (2.48)	3 (2.21)	
*IV*	357 (25.00)	312 (24.15)	45 (33.09)	
**The symptoms associated with HIV infection last 3 months, *n* (%)**				
Yes	141 (9.87)	122 (9.44)	19 (13.97)	0.092
No	1,287 (90.13)	1,170 (90.56)	117 (86.03)	
The last CD4^+^T cells (cells/μL), median (IQR)	438.50 (303.00, 604.00)	441.00 (302.00, 612.00)	380.50 (273.25, 566.75)	0.062
<350	474 (33.19)	420 (32.51)	54 (39.71)	0.162
350–500	408 (28.57)	369 (28.56)	39 (28.68)	
>500	546 (38.24)	503 (38.93)	43 (31.62)	
The last CD8^+^T cells (cells/μL), median (IQR)	625.00 (457.50, 887.00)	632.00 (459.00, 882.00)	660.00 (454.00, 956.75)	0.609
320–1,250	1,177 (82.42)	1,069 (82.74)	108 (79.41)	0.332
<320 or >1,250	251 (17.58)	223 (17.26)	28 (20.59)	

### 3.3 The knowledge and attitudes toward COVID-19 vaccination

For PLWH in this study, the most sources of COVID-19 vaccine information were government agencies (50.42%), followed by television or radio (40.20%), social media (35.64%), and medical staff (32.42%), while only a small percentage of information came from friends and families (18.42%) and others (16.45%).

In addition, when comparing the attitudes toward COVID-19 vaccination between the willingness and unwillingness to accept vaccination groups, we observed a significantly higher willingness to accept COVID-19 vaccination in PLWH who believe the vaccine is effective and safe and those who know at least one type of domestic COVID-19 vaccine (*p* < 0.001). However, PLWH who believe that vaccination will affect ART efficacy had a lower willingness to receive the COVID-19 vaccination (*p* < 0.001). There was no statistical difference in the attitude toward taking the initiative to consult the medical staff about the COVID-19 vaccination between the two groups (*p* > 0.05) (Table 3).

**Table 3:** Knowledge and attitudes toward COVID-19 vaccination of all participants.

Variables	All participants (*N* = 1,428)	Willing to receive COVID-19 vaccination (*N* = 1,292)	Unwilling to receive COVID-19 vaccination (*N* = 136)	*p*-value
**COVID-19 vaccination is effective**				
Yes	1,213 (84.94)	1,152 (89.16)	61 (44.85)	<0.001
No	28 (1.96)	14 (1.08)	14 (10.29)	
Uncertain	187 (13.10)	126 (9.75)	61 (44.85)	
**COVID-19 vaccination is safe**				
Yes	1,196 (83.75)	1,151 (89.09)	45 (33.09)	<0.001
No	19 (1.33)	6 (0.46)	13 (9.56)	
Uncertain	213 (14.92)	135 (10.45)	78 (57.35)	
**COVID-19 vaccination will affect ART efficacy**				
Yes	85 (5.95)	50 (3.87)	35 (25.74)	<0.001
No	651 (45.59)	644 (49.85)	7 (5.15)	
Uncertain	692 (48.46)	598 (46.28)	94 (69.12)	
**Consulting proactively vaccination information through medical staff**				
Yes	854 (59.80)	774 (59.91)	80 (58.82)	0.806
No	574 (40.20)	518 (40.09)	56 (41.18)	
**Know at least a type of domestic COVID-19 vaccine**				
Yes	1,319 (92.37)	1,212 (93.81)	107 (78.68)	<0.001
No	109 (7.63)	80 (6.19)	29 (21.32)	

### 3.4 The factors associated with the willingness to receive the COVID-19 vaccination

A total of 10 variables (*p* < 0.05) were presented in the multivariable logistic regression analyses. The results showed that PLWH were more unwilling to be vaccinated for those who were female (aOR 2.15, 95% CI 1.02–4.56) or fair/poor health status (fair: aOR 1.77, 95% CI 1.09–2.87; poor: aOR 3.01, 95% CI 1.03–8.86), those who had allergic history (aOR 2.07, 95% CI 1.25–3.45) and comorbidities (aOR 2.18, 95% CI 1.28–3.71), those who were unconvinced and unsure about the effectiveness of vaccines (unconvinced: aOR 6.71, 95% CI 2.01–22.46; unsure: aOR 2.62, 95% CI 1.48–4.63), those who were unconvinced and unsure about the safety of vaccines (unconvinced: aOR 6.01, 95% CI 1.63–22.17; unsure: aOR 4.38, 95% CI 2.49–7.69), those who were convinced and unsure about that COVID-19 vaccination will affect ART efficacy (convinced: aOR 25.42, 95% CI 9.64–67.01; unsure: aOR 7.56, 95% CI 3.25–17.59), those who do not know at least a type of domestic COVID-19 vaccine (aOR 3.59, 95% CI 1.85–6.97) (Table 4).

**Table 4:** Factors associated with the willingness to receive the COVID-19 vaccine.

Variables	Crude OR (95% CI)	*p*-value	Adjusted OR (95% CI)	*p*-value
**Sex, *n* (%)**				
Male	Ref.		Ref.	
Female	1.97 (1.22–3.47)	0.019	2.15 (1.02–4.56)	0.046
**Marital status, *n* (%)**				
Married	Ref.		Ref.	
Other (single, divorced, or widowed)	1.71 (1.18–2.48)	0.005	1.44 (0.88–2.36)	0.145
**Monthly income (RMB), *n* (%)**				
<2000	Ref.		Ref.	
2,000–5,000	0.61 (0.40–0.94)	0.026	0.68 (0.39–1.18)	0.168
5,001–10,000	0.79 (0.49–1.27)	0.328	0.75 (0.40–1.42)	0.380
>10,000	1.42 (0.71–2.83)	0.316	1.42 (0.55–3.69)	0.472
**Health status, *n* (%)**				
Good	Ref.		Ref.	
Fair	3.44 (2.34–5.08)	<0.001	1.77 (1.09–2.87)	0.022
Poor	14.46 (6.28–33.30)	<0.001	3.01 (1.03–8.86)	0.045
**Allergic history, *n* (%)**				
No	Ref.		Ref.	
Yes	2.61 (1.76–3.87)	<0.001	2.07 (1.25–3.45)	0.005
**Comorbidities, *n* (%)**				
No	Ref.		Ref.	
Yes	2.08 (1.40–3.09)	<0.001	2.18 (1.28–3.71)	0.004
**COVID-19 vaccination is effective**				
Yes	Ref.		Ref.	
No	18.89 (8.62–41.37)	<0.001	6.71 (2.01–22.46)	0.002
Uncertain	9.14 (6.13–13.64)	<0.001	2.62 (1.48–4.63)	0.001
**COVID-19 vaccination is safe**				
Yes	Ref.		Ref.	
No	55.42 (20.14–152.48)	<0.001	6.01 (1.63–22.17)	0.007
Uncertain	14.78 (9.83–22.22)	<0.001	4.38 (2.49–7.69)	<0.001
**COVID-19 vaccination will affect ART efficacy**				
Yes	64.40 (27.22–152.34)	<0.001	25.42 (9.64–67.01)	<0.001
No	Ref.		Ref.	
Uncertain	14.46 (6.66–31.42)	<0.001	7.56 (3.25–17.59)	<0.001
**Know at least one type of domestic COVID-19 vaccine**				
Yes	Ref.		Ref.	
No	4.11 (2.57–6.56)	<0.001	3.59 (1.85–6.97)	<0.001

Socio-demographic characteristics, health status characteristics and HIV-related characteristics, knowledge and attitudes toward COVID-19 vaccination were included in the regression according to the univariate analysis (*p* < 0.05), and willingness to receive COVID-19 vaccination as the reference group.

OR, odds ratio; CI, confidence interval.

### 3.5 The status and safety of the COVID-19 vaccination

**Figure 2:**
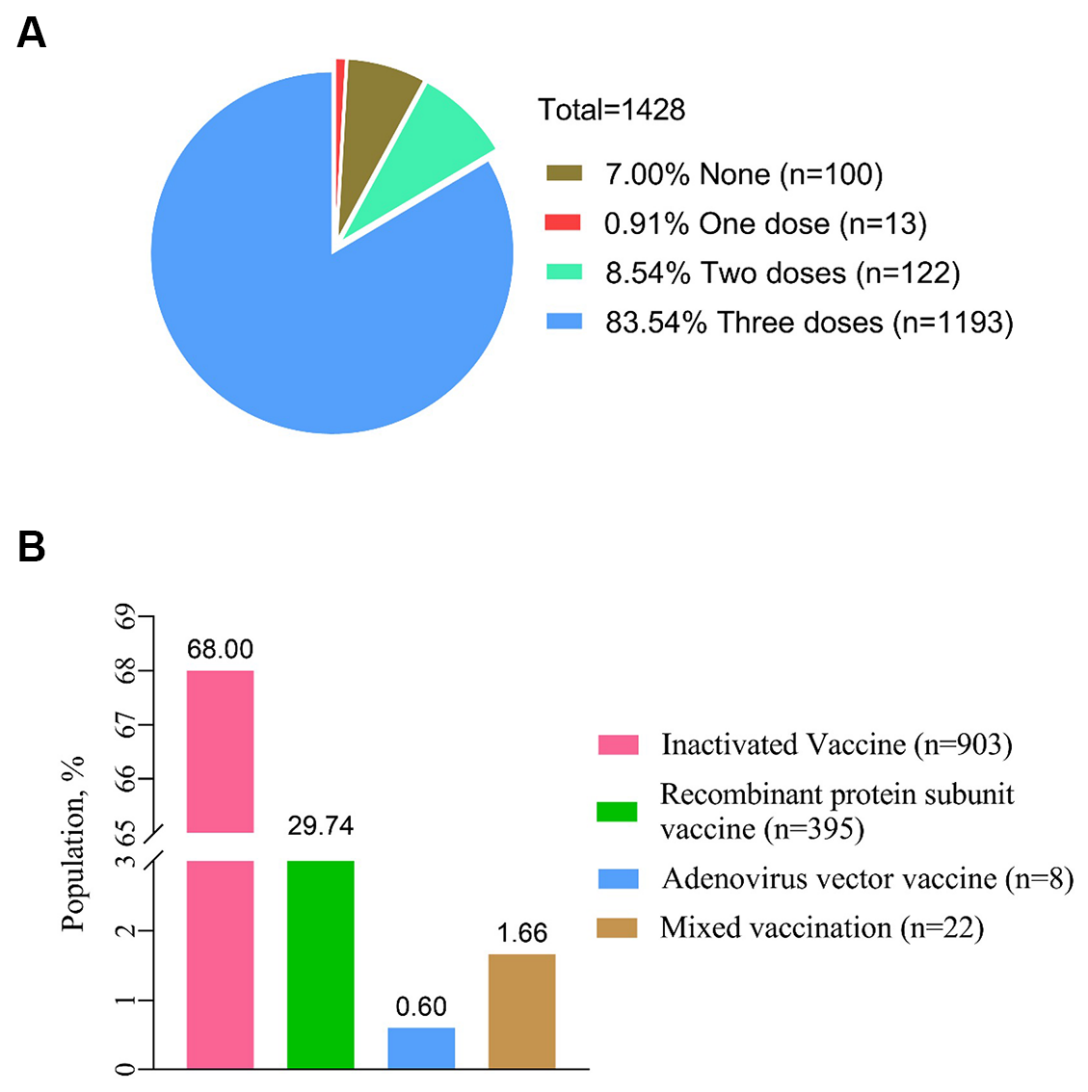
Status of COVID-19 vaccination. **(A)** Proportion of the different doses for COVID-19 vaccination (*n* = 1,428). **(B)** Status of vaccination with different types of COVID-19 vaccines (*n* = 1,428).

**Figure 3:**
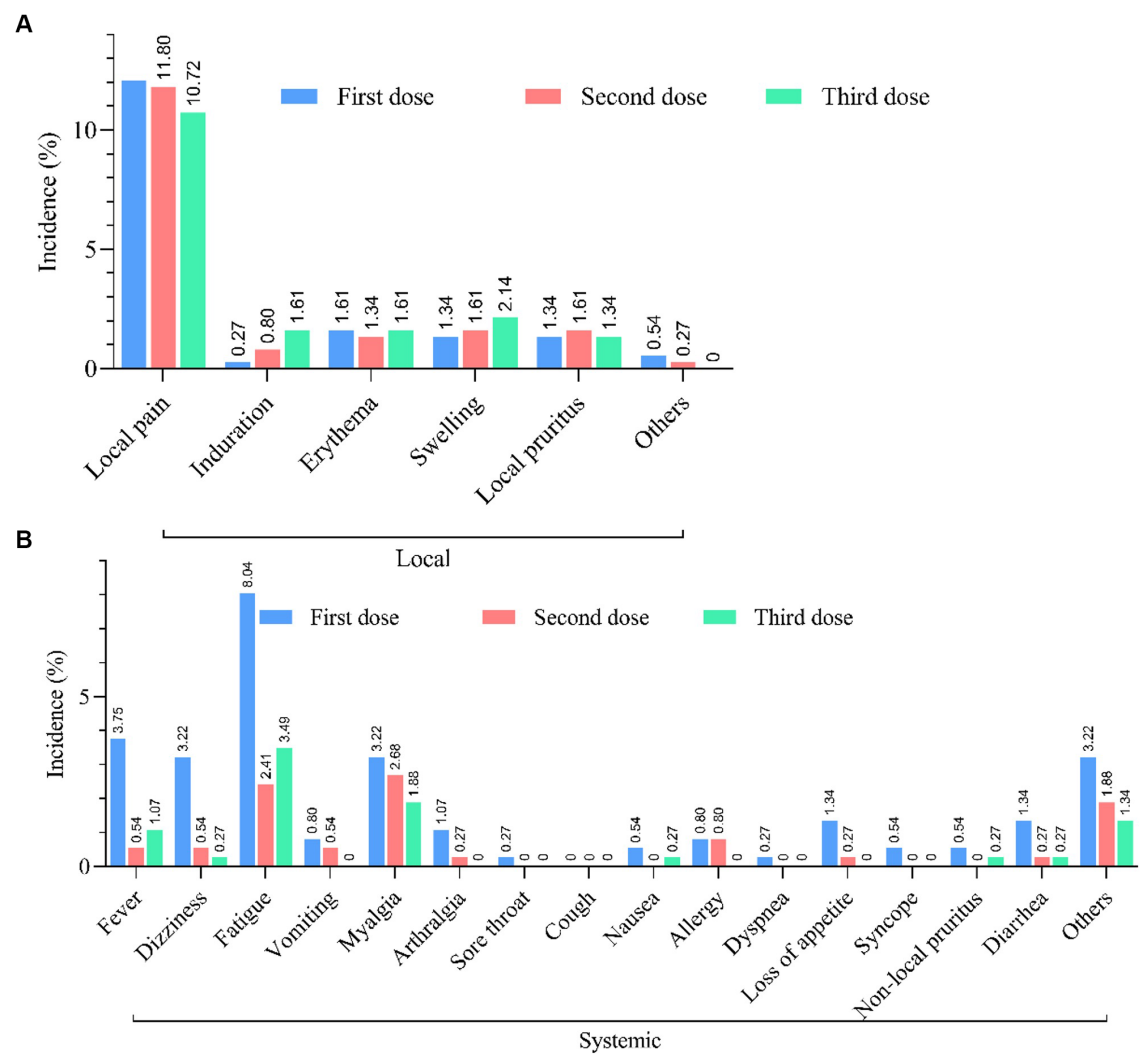
Safety of COVID-19 vaccination. **(A)** Incidence of local and systemic adverse reactions reported within 7 days after each dose of COVID-19 vaccine in PLWH (*n* = 213). **(B)** Incidence of systemic adverse reactions reported within 7 days after each dose of COVID-19 vaccine in PLWH (*n* = 213).

In a total of 1,428 PLWH, 1328 (93.00%) have received at least one dose of the COVID-19 vaccine, and the largest proportion (68.00%) have received inactivated vaccines. After being vaccinated against COVID-19, 213 PLWH (14.92%) reported at least one adverse reaction within 7 days (Figure 2). The most common local and systemic adverse reactions were local pain (12.06% first dose, 11.80% second dose, and 10.72% third dose) and fatigue (8.04% first dose, 2.41% second dose, and 3.49% third dose), respectively, and no serious adverse events have been reported (Figure 3).

## 4 Discussion

Vaccination is one of the most effective measures to end the COVID-19 pandemic. According to an expert recommendation on COVID-19 vaccination for PLWH released from China in July 2021, PLWH could also be vaccinated after their HIV-related condition was assessed (26). In addition, authoritative guidance for COVID-19 and people with HIV developed by the Guideline Working Groups of the National Institutes of Health (NIH) Office of AIDS Research Advisory Council (27) suggested that PLWH, regardless of CD4^+^T cells or viral load, should receive COVID-19 vaccination since the potential benefits outweigh the potential risks. Even so, 31.6% of PLWH reported COVID-19 vaccination unwillingness worldwide (19). Therefore, it is important to understand the willingness of PLWH to receive the COVID-19 vaccination and the factors influencing it.

In the present study, we found a high willingness for COVID-19 vaccination (90.48%) among PLWH in Shijiazhuang, which was almost consistent with the finding in Turin, Italy (92.4%) (28). However, it was significantly higher than that of the general adult population in China (60.4 and 82.6%) (29, 30) and around the world (68.4 and 71.5%) (19, 20). This proportion was higher than that of PLWH in China (57.2%), the United States (83.8%) (24), South Africa (57%) (31), and Northern Nigeria (46.2%) (32). In summary, this study reflected that COVID-19 vaccination willingness among PLWH is relatively high in Shijiazhuang, China. The results of this study should be viewed with caution. We conducted this study on 15 June 2022, which was 17 months later than the launch of the national free vaccination policy (33). At this time, studies in China and Brazil have shown a comparable immune response and safety between PLWH and healthy individuals in response to inactivated COVID-19 vaccine (23, 34), and similar results were also observed in adenovirus vector and messenger RNA (mRNA) COVID-19 vaccines (35, 36), which may be one of the reasons affecting the higher willingness to COVID-19 vaccination. In addition, the propaganda of government agencies plays an important role as it contributes the highest percentage (50.42%) of information sources in COVID-19 vaccines, according to our findings.

The results of multivariable logistic regression analyses indicated that some variables in socio-demographic characteristics, health status characteristics, and knowledge and attitudes toward COVID-19 vaccination were significantly associated with vaccination willingness. For example, the female PLWH had lower COVID-19 vaccination willingness than the male, which was consistent with a prior study in older PLWH (≥50 years) in the Coachella Valley (37). PLWH with fair/poor health status, an allergic history, and comorbidities also reported lower willingness, which suggests that poor health conditions are likely to affect PLWH’s willingness to be vaccinated. A study of the PLWH in French reported that those who are worried about their health status and underlying diseases were more likely to accept COVID-19 vaccination (38). Another study in China also found that PLWH with comorbidities were more willing to be vaccinated (39). An earlier study (from Wuhan, China) showed that PLWH with comorbidities had a higher willingness to receive the COVID-19 vaccination (39), which was diametrically opposed to the results of our study. The reasons for this phenomenon may be related to the late date of our study and the different geographical areas.

In addition, knowledge and attitudes toward COVID-19 vaccination were important factors influencing COVID-19 vaccination willingness. PLWH who were unconvinced and unsure of the effectiveness and safety, those who are convinced and unsure about whether COVID-19 vaccination will affect ART efficacy, and those who do not know at least a type of domestic COVID-19 vaccine had lower COVID-19 vaccination willingness, which is consistent with the findings in China (23, 40), the United States (41), and France (38). The phenomenon is common because vaccine-specific issues are the most frequent determinants of unwillingness to be vaccinated. In addition to COVID-19 vaccines, similar phenomena also existed in other types of vaccines served among PLWH. A study in France has shown that fear of expected effectiveness and adverse effects were the most frequent reasons for refusing vaccination for *Streptococcus pneumoniae*, influenza, tetanus, and chronic hepatitis among PLWH (42). This suggests that subjective attitudes among PLWH have a strong influence on willingness to be vaccinated because our research found that none of the HIV-related characteristics had a significant effect on the COVID-19 vaccination willingness, including time living with HIV, mode of HIV transmission, clinical stage, the symptoms associated with HIV infection the last 3 months, and the last CD4^+^/CD8^+^cells. Therefore, in order to further improve COVID-19 vaccination coverage, it is necessary not only to strengthen research on the efficacy and safety of vaccines but also to emphasize the publicity and popularization of knowledge about vaccination among PLWH.

According to the finding, 93.00% of PLWH have received at least one dose of the COVID-19 vaccine, which is higher than that among PLWH in the United States (64%) (43) as well as in the Middle East and North Africa region (19.3%) (19). The vaccination rates are not comparable between regions due to the fact that the above study was conducted in early 2021. Previous studies have proven that COVID-19 vaccines have a good safety profile among PLWH (44, 45), and similar results were obtained in our study.

This study has several limitations. Because the cross-sectional study design was unable to determine causality, we can only describe associations between COVID-19 vaccination willingness and influencing factors. We recruited an opportunistic sample, which may affect the generalization performance. In addition, information bias is inevitable because part of the data was obtained through online methods, and the health status of patients is subjectively judged by patients themselves according to their own physical conditions, which have not been collected using validated measures. The in-person questionnaire may put limits on the “sincerity” of the answers, but we did not analyze whether there are any differences in willingness according to the way of survey administration. Nevertheless, rigorous data examination was set to exclude ineligible participants and ensure data quality. Another limitation of our study is that we neglected to design-related questions to assess the psychological state of the subjects, which is also a strong predictor of vaccine hesitancy. COVID-19 vaccination willingness will be changing over time due to the multiple influencing factors, so a study considering more aspects will be performed to certify our result.

## 5 Conclusion

In conclusion, our study showed that PLWH in Shijiazhuang reported a relatively high willingness to receive the COVID-19 vaccination. There is a lower willingness to receive COVID-19 among PLWH who are female or have a fair/poor health status, those who have an allergic history and comorbidities, those who are unconvinced and unsure about the effectiveness and safety of COVID-19 vaccination, those who are convinced and unsure about whether COVID-19 vaccination will affect ART efficacy, and those who do not know at least a type of domestic COVID-19 vaccine. Consequently, more tailored policies or guidelines from the government should be implemented to enhance COVID-19 vaccination coverage among PLWH.

## Data availability statement

The original contributions presented in the study are included in the article/supplementary material, further inquiries can be directed to the corresponding authors.

## Ethics statement

The studies involving humans were approved by the study was approved by the Ethics Committee of the Fifth Hospital of Shijiazhuang (2022-017-1) and the Medical Ethics Committee of Peking Union Medical College Hospital (JS-2156). The studies were conducted in accordance with the local legislation and institutional requirements. Written informed consent for participation was not required from the participants or the participants’ legal guardians/next of kin in accordance with the national legislation and institutional requirements.

## Author contributions

XZ: Formal Analysis, Investigation, Methodology, Software, Visualization, Writing – original draft, Writing – review & editing. HZ: Data curation, Formal Analysis, Investigation, Methodology, Software, Visualization, Writing – review & editing, Writing – original draft. LW: Investigation, Project administration, Supervision, Writing – review & editing. YLiu: Formal Analysis, Methodology, Resources, Writing – review & editing. XG: Investigation, Resources, Writing – review & editing. CL: Investigation, Writing – review & editing. XL: Investigation, Supervision, Writing – review & editing. BL: Investigation, Writing – review & editing. HL: Investigation, Methodology, Writing – review & editing. YiL: Project administration, Writing – review & editing. QC: Resources, Writing – review & editing. HG: Investigation, Project administration, Resources, Writing – review & editing. FF: Conceptualization, Project administration, Resources, Supervision, Writing – review & editing. YoL: Conceptualization, Funding acquisition, Investigation, Methodology, Project administration, Resources, Supervision, Writing – review & editing. ED: Conceptualization, Funding acquisition, Methodology, Project administration, Resources, Supervision, Writing – review & editing.
